# The Hospital. Nursing Mirror

**Published:** 1898-06-11

**Authors:** 


"The Hospital" Nursing Mirror.

Being the Nursing Section of "The Hospital."

[Contributions for this Section of "The Hospital" should be addressed to the Editor, The Hospital, 28 & 29, Southampton Street, Strand, London, W.C., and should have the word "Nursing" plainly written in left-hand top corner of the envelope.]

### News from the Nursing World.

### LONDON SCHOOL NURSES' SOCIETY.

The new development in the work of the district nurse, known as the London School Nurses' Society, will effect much good, both directly and indirectly. Directly, because the petty ailments of the children attending elementary schools will be detected and nipped in the bud by experienced and trained women; indirectly, by raising the standard of general health, and by tracing graver maladies to their sources before they have time to become firmly established. The committee, of which the Hon. E. Lyulph is the chairman, and Miss Honnor Morton the hon. secretary, are now appealing for £150 to enable them to engage a visiting nurse for Hoxton, Tower Hamlets, and Southwark at once. It is estimated that the cost will be about £50 a school, and that one nurse can take charge of more than one school. Contributions should be sent to the Hon. Treasurer, 89, Harley Street, W.

### THE MEDICAL, SURGICAL, AND HYGIENIC EXHIBITION.

For four days the Queen's Hall, Langham Place, W., was the scene of an exhibition in the highest degree useful and instructive to medical men, nurses, and those who deal in medical specialities. There were to be found the latest inventions in instruments and appliances side by side with the newest drugs, and the most improved methods of preparing them. Stalls were devoted to the numerous foods, and also to medical literature. Many of the visitors were interested in tasting as well as seeing some of the samples collected for their inspection, and their amusement was well catered for by numerous concerts. There were a large number of nurses amongst them, generally on business intent, with their hands full of leaflets and their attention fixed on some novelty or other most applicable to their own immediate requirements. The exhibition, we hear, is to be a yearly occurrence.

### THE SOCIETY OF CHARTERED NURSES.

The founders of the Society of Chartered Nurses have every reason to congratulate themselves on their venture. Although the second anniversary has barely passed the society is widely known and appreciated, and nurses are sent all over England. Miss Jackson, the secretary, has adopted all the recent improvements in the business arrangements. A telephone has been brought into her office, and a speaking tube fixed for night work. She has engaged a shorthand clerk for some hours in the mornings and a chartered accountant to supervise all accounts, and yet her own time is fully occupied. Last year's work and receipts were double those of the first, and nearly one thousand cases were supplied with nurses. The staff will be raised to 100 at the next committee meeting. Much of the success is undoubtedly due to the care with which the nurses are chosen, preference being given to those of longer experience rather than to the more recently qualified. Most of the nurses have joined the Royal National Pension Fund, and as the work is steady their average earnings are high. During the first two months of the present year there was an unprecedented demand for nurses, and recently there was only one nurse on the staff disengaged.

### WHO SHOULD BELL THE CAT?

When things go wrong censure usually falls upon those in authority; but the question often arises, "Who should bell the cat?"—in other words, who should administer chastisement? The Guardians of Belfast Union Workhouse richly deserve a severe lesson, for the inadequate numbers on the nursing staff has undoubtedly caused a child's death. The inquest brought to light the fact that Nurse Nugent and one probationer had charge of 194 sick from nine o'clock in the evening to seven the next morning; and, although the coroner stigmatised this as an "outrageous state of affairs," and the jury added a rider to their verdict to the effect that the "nursing staff is quite insufficient," they declared also that "they attached no blame to anyone." In regard to this workhouse, Mr. Robert Agnew, the Local Government Board's Inspector, recently reported that the quality of the food is unsatisfactory; that the main infirmaries are overcrowded; that twelve nurses and more probationers are required to bring the nursing staff to a state of efficiency; and that pauper orderlies are employed in direct contravention of the Board's order. Under these circumstances, it is time for the Local Government Board to bring the mismanagement home to the offenders, and institute a thorough reform.

### WESTMINSTER NURSING HOME AND WESTMINSTER HOSPITAL.

We hear on excellent authority that the appointment of matron to the Westminster Hospital and Westminster Nursing Home, vacant by the resignation of Miss Pyne, is not finally settled, and it appears probable that two matrons will be appointed, one to the hospital and one to the nursing home. There can be no doubt that the combined work is a very severe tax on one individual.

### A FITTING MEMORIAL TO GENERAL GORDON.

When Khartoum is once more under civilised rule, an effort will be made to erect a suitable memorial to General Gordon in his death place. Dr. King, the Bishop of Lincoln, already entertains the hope that it will take the form of a hospital, staffed by Christian doctors and nurses, who will thus fittingly carry on and commemorate the hero's life-work.

### THE LONDON SPECTACLE MISSION SOCIETY.

On June 1st this society, which quietly pursues a useful course, opened a new branch at St. Benet's, Mile End Road. Its aim is to provide poor working folk, seamstresses, tailors, and the like, with good spectacles. It deals only with those whose sight is failing in a natural way, and not with those suffering from defective vision requiring the assistance of an oculist. The spectacles are of English make, of good glass in steel frames. Miss Waring, daughter of the originator, is hon. secretary to the society, which has been managed now for some years by a committee. The Bishop of London, Canon Duckworth, and other well-known gentlemen are interested in it. Subscriptions are invited, and may be sent to Miss Waring, 197, Sutherland Avenue, London, W. Five shillings entitles the contributor to recommend four cases.

### MELBOURNE NURSES.

The fourteenth annual meeting of the Melbourne District Nursing Society, held in the Town Hall on March 10th, brought to light a grave state of affairs. Since the beginning of the year the colony, both in town and country, has suffered severely from scarlet fever and measles, whilst typhoid has been continuously prevalent. It is sincerely to be hoped that the new system of drainage at Melbourne, now rapidly reaching completion, will exercise a beneficial effect on the public health, which is far below a normal standard. Unfortunately, not only sickness, but poverty has been widespread, and at the moment when the need is, from this fact, greatest, the society has decided to dispense with the services of their maternity nurse.

### MALCONTENTS.

Many nurses start in their careers with their faith pinned on their certificates, and are bitterly disappointed when the piece of parchment fails to prove the "open sesame" before which all bars fall. We have heard of a nurse with excellent certificate and unimpeachable character being dismissed from a Co-operation of Private Nurses, and vehemently questioning the right of her committee to send her away. "I am a good nurse," she said, "therefore you cannot discharge me." Her fault chanced to be one that cannot be too strictly guarded against, though is not uncommon. Immediately upon taking charge of a new case it was her custom to retail all her private affairs and grievances, harping incessantly upon these topics until those compelled to listen to her tales of woe were thoroughly wearied. Finally, resenting some strictures of the medical man whose patient she was attending, she threw up the case, and departed without observing any of the usual formalities, becoming highly indignant when the society to which she belonged declined to find her another. Such women bring discredit not only upon their own nursing home, but also upon the calling. They quarrel with everyone, and, although backed by splendid certificates, arrive finally in the ranks of the malcontents who have, as a rule, little to do but talk.

### MAINTAINING THE ROLL CALL.

It is easy to call spirits from the vasty deep; the difficulty lies in compelling them to come. A lady of some prominence in the nursing world not so long ago called upon all matrons to withdraw from the Royal British Nurses' Association in consequence of the effect of the new bye-laws. The result measures the power of the call, and it is a fact not without significance that only seven resignations have been received by the secretary for some time past, and these not from matrons. Of these seven nurses two had not paid their subscriptions for two years, two for three years, and three for one. The resignations were, therefore, from persons who had practically ceased to be members. In the last number of the *Nurses' Journal* there is a list of applicants for membership more than covering the loss. Therefore, notwithstanding the troublesome times through which the Association has been passing, the roll call of members is fully maintained.

### GLOUCESTER DISTRICT NURSING SOCIETY.

At the annual meeting of the Gloucester District Nursing Society several items of a gratifying character were reported. The superintendent has been awarded the silver badge of Queen Victoria's Jubilee Institute for Nurses. The nurses are available for all the sick within a five mile radius of Gloucester Cross. The balance of the subscriptions sent to the Small-pox Committee, amounting to £1,461, has been invested, and the yearly interest applied to the maintenance fund of the society. There is a prospect of speedily establishing a permanent nursing home. The Guardians have contributed £150 towards expenses; and last, but by no means least, the adverse balance of the previous year of £189 has been reduced to £29. The committee is endeavouring to obtain a yearly collection in places of worship of all denominations and secure an annual income to cover all expenses.

### SHORT ITEMS.

The income of the Newlyn Nursing Society for the past year was £60, and the expenditure exceeded that sum by £8. The nurse has paid 2,311 visits. The constitution of the association is so little known that many people supposed it to be a Government institution, and the suggestion of the Rev. H. Adams, to send circulars to the people in the neighbourhood explaining its position and asking for subscriptions, was adopted.—At the annual meeting of the Ham and Petersham District Nursing Association, Dr. Goodman proposed to withdraw from his office of hon. treasurer, in case people might come to think that the nurse of the association was in his own employ. He was unanimously requested to retain his post, as Nurse Wing was well known to be "everybody's nurse." She is, moreover, so generous and popular that her salary was increased by £5 per annum.— Miss Peter, Inspector of Nursing of Q.V.J.N.I., writes, as follows, of the Buntingford. Nursing Association: "I had a most satisfactory visit to Buntingford. Nurse Hawkes' work is very good, and she seems happy and comfortable."—The district nurses of the Doncaster and District Nursing Association have been removed from 56, Hallgate to 15, Christ Church Road, Doncaster.—A sum of £14 was collected in the streets of Glasgow for the nursing association, on the occasion of a parade in fancy costume by the Lockerbie Cycling Club—Miss Maud Tweed, of the Nurses' Training School, Frederick Street, has obtained the appointment of fever hospital nurse under the Ballymoney Board of Guardians.—The Local Government Board requires the York Board of Guardians to appoint a superintendent nurse. The Guardians had requested to be allowed to dispense with this officer.—Several manufacturers in Birmingham have contributed gifts of furniture to the new nurses' home at No. 94, Moseley Road, acquired by means of the Diamond Jubilee Fund. There is money in hand to purchase a second home, and the committee of the association hope that other manufacturers, who have not yet contributed, will follow so excellent an example.—The Finance Committee of the Nursing Home, Shanghai, are increasing the fees charged for the services of the municipal nurses.

### Lecture to Nurses.

### ON PREPARATION FOR AND AFTER TREATMENT OF CASES OF ABDOMINAL SECTION.

By E. Stanmore Bishop, F.R.C.S.Eng., Surgeon to the Ancoats Hospital, Manchester.

(*Concluded from page* 91.)

If the vagina requires preparation, besides all this, something more is required. All hair must be removed by shaving, since the sebaceous follicles are particularly numerous around hair bulbs, and it is impossible to get at them unless this is done. Then the same preparation as for the skin elsewhere must be carried out on all the external parts with extra care, since bacteria are peculiarly liable in the half-soddened epithelium at the bottom of any folds of skin. The interior is irrigated, preferably with creolin, 1 in 300, and then with pure water, and this irrigation must be thorough, two fingers of the nurses' left hand being introduced to straighten out the folds of mucous membrane and to see that the sterilising fluid finds its way into every part. I prefer, when I can get it done, to have the part first washed out with soap and water, because the alkali of the soap dissolves the mucus always found in a cavity lined by mucous membrane, and which lying over this protects it from the action of the watery solution of creolin or biniodide. When all has been done, a little cotton wool is placed just inside until the next irrigation. About three irrigations of a couple of quarts each are usually necessary at intervals of twelve hours, and the last should be on the operating table or just before. So much for cleansing the outside of the patient.

Can we cleanse the inside? Yes, but not so completely, and what we do we do not so much for that reason as for another, to which I will in a moment draw your attention.

When a patient has undergone abdominal section, there are three main risks she has to run, and they are these: (1) Secondary hæmorrhage, which risk is practically over in twenty-four hours, and which is, if it occurs, due to my fault, and so does not concern you, except so far that you ought to know how to recognise it, and so be able to warn those whose business it is. I will speak of this later. (2) Shock and collapse, which come on usually as the patient is ecovering from chloroform, and are due partly to the length nd kind of operation, which is not your affair, and partly to undue chilling of the body during the time it is on the table, which in great measure is. Now chilling we try to avoid: (1) By keeping the doors and windows shut. This we do also to prevent any eddies of dust rising from the floor. I want you to remember this, because occasionally I see nurses entering and leaving the theatre whilst work is going on. Everything anyone can possibly want should be in the room before we begin; and even if you are hungry, and I have no doubt I have tried your patience in that respect more than once, you should try and restrain your appetites in the interests of the patient until the work is over and the body once more warmly covered. I guess your sufferings by my own, but the patient should always come first. (2) By having the temperature of the room at a good height. It should never be less than 70 deg., and therefore the fire should be seen to, so that there is no fear of its dying down too far and requiring fresh coal, which always makes some dust and spoils your hands when once prepared. (3) By warming the operation table itself. Hot water should be run through the tubes until they are hot. The metal plates should be taken off and heated. When blankets are laid upon them it takes some time before the heat penetrates, and a good deal is lost by radiation, time, &c. (4) By arranging blankets, &c., over chest, legs, and thighs. In private work I have found much benefit from long cycling men's stockings, into which a layer of cotton wool has been quilted. Blankets should not merely be thrown over, they should be tucked in all round and underneath. If you are cold in bed is it not your first endeavour to tuck in the bed-clothes around you, so that no little cold draughts of air can reach your skin? and although the patient is unconscious and so cannot complain, the cold air has precisely the same effect upon her as though she were awake, and perhaps somewhat more, since her vitality is lowered at the time. (5) When the patient is returned to bed, by hot bottles, warm flannel wrapped round the head, and warm bags and bottles around her, which, however, must be watched lest they scorch the skin. (6) By the injection of rectal enemata containing brandy and hot saline solution, which there is reason to think is more effectual than beef-tea in combating shock. The latter, shock, our second risk, is also combated by the previous coaxing of the patient to drink freely during the forty-eight hours previous to the operation of as much fluid as she can manage, with the exception of some four hours immediately before. Professors Cobbett and Roy have made some experiments which show that any operation involving the opening of the abdomen, and especially interference with the intestine, is followed as a matter of course, by overfulness of the abdominal veins, into which much, if not most, of the blood in the body finds its way. These veins lose what is called their tone, and passively dilate. This passes off in the course of a few hours, but meanwhile the other vessels, and especially the heart and cerebral vessels, must be kept with a reasonable amount in them, and they absorb whatever fluids the tissues have to spare. If there is a fair amount, the danger is tided over; if there is not there comes a time when it is exhausted, and if the drain into the abdominal veins has not yet ceased the heart and the vessels conveying blood to the important nerve centres at the base of the brain become empty and severe collapse, possibly ending in death, ensues. But you will say, why not give the patient liquid afterwards? We hear that you keep your patients for 24 and 48 hours after without any liquid by the mouth. Surely this is inconsistent; and the answer to that brings me to the third risk, which is present from 12 to 24 hours after operation, and the danger of which cannot be said to be over until after the fifth day, and that is septic poisoning. I do not say septic peritonitis, because peritonitis is only Nature's effort to localise the poison, and is our danger signal showing that poison is present.

The peritoneal cavity is one great absorptive surface. It is computed that the whole surface is equal to that of the entire skin and that it is able to absorb 8 per cent. of the body weight in 24 hours. Now we have seen that bacteria require for their development heat and moisture. Let us suppose that in spite of all our care a few spores have found an entrance. If they can lie quiet in a small pool of fluid they will develop rapidly. I forget how many one can generate in 24 hours, but it is many thousands; I think Richmond says sixteen millions. We cannot deprive them of warmth unless we freeze our patient, but we can of moisture. Through the whole of the cavity runs what is practically a living sponge, the small intestine, which, alternately dilating and contracting, sucks up by its peritoneal surface all the moisture present. Especially will it do this if it contains in its interior but little fluid. The fluid you have given beforehand is by this time all, or most of it, in the tissues, and can only reach the intestine again in a roundabout way. So long as it and the membrane over the interior of the abdominal wall absorbs, so long will the cavity remain dry, and the bacteria are hurried along into the lymphatic vessels and glands by their natural enemies, the wandering leucocytes, the natural volunteer defenders of the body, which devour them alive. Should they remain quiescent long enough, they secrete poisons—toxins, as they are technically called, which paralyse the action of the gut, and it lies inert and motionless—so that it is all important at the earliest period to enlist the help of osmosis, as it is called, the tendency of fluids to pass through animal membranes, and by keeping the interior of the bowel as dry as it can be got, to render the flow of fluid from without the bowel inwards. As soon as possible, too, we like to get evidence that the bowel is well alive and contracting as I have described, and so we give drugs—calomel, seidlitz, mist. alba, &c.—so that it may be thereby stimulated to keep up in active force such contraction. So we eagerly look for the first passage of flatus, and assist its escape by the rectum tube. The reason for the use of the latter is this. Patients after an operation are weak; their tissues share in this weakness. The muscular walls of the intestine, being part of such tissues, necessarily are likewise weak. They may be strong enough to force on their contents as long as there is no opposing block, but when these reach the sphincter, they are not quite strong enough to overcome its contraction. Then gas accumulates, and it stretches the already weak muscular fibre in the bowel wall. Now it is a universal law that muscular fibre acts less strongly the more it is stretched, and you can see that a point would soon be reached when the force of the dilating gas would entirely overpower the weak muscular wall, and a kind of paralysis of the gut would be produced. This is precisely what we dread, and therefore we dilate, and keep dilated, the sphincter by the passage of the rectum tube, and so allow the gas to escape as soon as it reaches this point. The escape of the flatus assures us that the gut is not paralysed, but is working, and it relieves the internal pressure, and so allows the bowel to act at most advanage until it has ecovered its full tone, when it no longer requires our assistance.

Now, if you have followed my explanation, and I have been fortunate enough to make it clear to you, you will see the reason for our previous preparation of the patient, and for most of the after-treatment. You will see why I advise that at every abdominal operation, every nurse who takes part, however little, should (1) See that everything is ready, assist the patient on to the table, and see that she is warmly covered and protected against all chill. (2) First, do anything she may have to do which necessitates the touching of things which are not themselves sterile. (3) Remove all cuffs, and turn up her sleeves to the elbow, so that they may by no possibility touch anything used in the operation itself. (4) Cut her nails short. (5) Scrub hands and arms for three minutes with soap and hot water, paying special attention to her nails. (6) Scrub again with biniodide solution in spirit (1—500) for two minutes. (7) Soak hands and arms in a warm watery solution of biniodide (1—1,000) for two minutes; this is done to wash off the spirit which otherwise would make the skin unpleasantly dry. (8) Avoid wiping her hands, but work with them wet. (9) After sterilising most carefully avoid touching anything in the least doubtful until the operation is over.

One word as to the signs of secondary hæmorrhage. The most important are: (1) Restlessness and gasping; (2) loss of colour in lips; (3) quickened and quickening pulse. Should these or any of these occur raise the foot of the bed, wrap the head in hot flannel if this has not already been done, and send for the house surgeon.

### Novelties for Nurses.

AT MESSRS. GARROULD'S,
150, Edgware Road, London, W.

Messrs. Garrould's new catalogue deserves more than a passing glance, so very complete is it as well as up to date. The excellence and variety of nurses' uniforms supplied by this firm has long since become a household word, so we will pass them by, merely drawing attention to some dainty designs in cap strings, some of which are frilled and others finished off with a neat hemstitched border that is eminently suitable and becoming for out-of-doors wear. Invalid and obstetric corsets have received special attention, with the result that a most comfortable and seasonable article is now placed within reach of our readers. A special corset designed for ladies who cannot bear pressure of any kind is sure to be in great demand; it is soft and comfortable, being finished only with bones at the back and sides, and only costs 10s. 9d. The new obstetric corset is a very ingenious contrivance. The sides are elastic, and there are lacings over the hips and in the front. There is also another of somewhat less elaborate design, which laces at the sides and is made in two qualities at 7s. 9d. and 14s. 9d. respectively. The "Nurses' bag," model 81, is a novelty that will bring joy to the heart of every nurse engaged in district work. It is made of dull black cowhide with covered frame and washable lining. It contains all the necessary requisites, and its price fitted complete is 27s. 6d. If preferred it may be had unfitted, and the purchaser can choose her own fittings according to taste. Model 95 is on a rather larger scale, and is one of the most complete bags we have ever seen. It really seems within its limited space to contain everything that a nurse can desire, so compactly is each article arranged for. The price of this treasure when fitted is only 47s. 6d., and is a marvel of cheapness. A new bath thermometer strikes one as a great improvement on those hitherto in use, and the size admits of it being easily stowed away in a convenient corner. The Cleerd Thermometer is another special design, its chief advantage being that the tube and bulb being raised it can be read without difficulty from a distance. When we consider the trouble there often is in clearly distinguishing the index in existing samples, we can the more readily appreciate the utility of this arrangement. The choice in instruments is very wide, and we desire to draw special attention to the scissors, which are excellent, both in quality and shape. Model 110, with flat blades, are most workmanlike and practical. They are to be had in four sizes. One very excellent departure we are pleased to notice, and that is the establishment of telephonic communication between the vast emporium and the public call offices. Any nurse wishing to give instructions for the immediate supply of any sick room or other requisite has only to go to the nearest call office and have her wants attended to, the charges being for three minutes' conversation in London or suburbs 3d., and for 100 miles or part of 100 miles from London 6d. The tea-room still remains an attraction, and daily becomes more largely patronised by nurses from all parts.

### Appointments.

MATRONS.

The Park Fever Hospital, Hither Green.—On June 4th Miss Ethel Jessie Atkins, matron of Cuddington Hospital, Banstead Downs, was appointed Matron of the Park Hospital. She was trained, and was afterwards staff nurse at St. Bartholomew's Hospital, London. She then became successively charge nurse of the South-Western Hospital, Stockwell; sister at the City Fever Hospital, Birmingham; and assistant matron of the Western Hospital, Fulham.

### Nursing in Paris Hospitals.

### B.—THE TRAINING OF NURSES.

### VI.—THE SCHOOLS FOR MIDWIVES: CLINIQUE D'ACCOUCHEMENT.

THE second governmental school of midwifery in Paris is the Clinique d'Accouchement, in the Rue d'Assas, near the Luxembourg Gardens, and not very distant from La Maternité. Although in an establishment under the control of the Assistance Publique, the school itself is attached to the Medical Faculty of the University of Paris, or, in other words, the Sorbonne, as it is familiarly called.

The School of Midwifery was established in August, 1879, and, like everything else connected with the University, the instruction itself is free, but certain fees have to be paid for examinations and diplomas. These fees consist in some cases of one sum of 130 francs, and in others of two sums of the same aggregate amount, divided into 50 francs for first examination and 80 francs for the second.

Students are admitted between October 1st and October 15th of each year, and must be at least nineteen years of age. There seems no maximum age limit as at La Maternité. As at La Maternité, a series of certificates are required, but an up-to-date provision is inserted for cases of divorced women to produce the documents relating to the divorce case.

Further qualifications are required of candidates for the diploma of first-class midwife. The second-class diploma is given at the end of the first year, after the annual examination is satisfactorily passed. For the first-class diploma, either the second year at the Rue d'Assas must be followed or the candidates must be already second-class midwives from La Maternité, or from provincial or private schools, producing certificates to this effect.

Special provision is made for exempting foreign students from the requirements of producing professional certificates. They are only required to pass the entrance examination.

The examinations at the end of the first year's course are concerning anatomy, physiology, and elementary physiology. The examination at the end of the second year's course for first-class certificates is concerning theoretic and practical midwifery.

Both at La Maternité and in the Clinique, whenever the surgeon has to be called in to a childbirth, either by day or by night, all students are summoned to the bedside for the special instruction.

At both schools, beside the regular annual examinations, special examinations can be ordered at any time, when students who are found too much below the required standard can be discharged from the courses.

When I visited the Rue d'Assas this year, the number of students pursuing the course of midwifery was 63. The medical students, besides the Rue d'Assas, have a special clinic of childbirth at the Baudelocque Hospital, adjoining La Maternité, where all pregnant women are admitted at all times free; but there is no midwifery school attached to the establishment, such a school naturally being superfluous with another school next door to it. Surgical students are allowed to practise at the Baudelocque, but not at La Maternité or the Rue d'Assas.

Probably no part of the nurse-training in the Paris hospitals is on such a solid footing as that of the midwives in these two schools. The case for the claims of modern pre-eminence was very eloquently put by the learned doctor of La Maternité, Dr. Charrin, in his address at the last distribution of diplomas.

A few of his remarks of special interest I will quote, as stating the present spirit of the administration of midwifery training. Referring first to the defects of olden times, he said: "Those who preceded you used to hear of miasmas, of contagion of peccant matter, even spirits of animals. Anyhow, they and their contemporaries were ignorant of the solid, liquid, or gaseous nature of the infectious agents. They were ignorant of their origin, habitats, or the nature of their life, their modes of action, reproduction, transportation: consequently they were obliged to renounce the rational knowledge of the processes capable of protecting the economy against these fatal aggressions."

Contrasted to this, the doctor described the method of to-day thus: "More fortunate than your predecessors, by the light of the positive data of experimental methods which admit only that which is understood, you grasp little by little what are all these mysterious existences, capable of producing the most terrible accidents! You know how they are evolved, how they are cultivated, and—most important point—how they are destroyed! You know what conduct to follow to prevent their penetration into us, and also how to force out those which have begun to introduce themselves without, however, having succeeded in slipping into the general circulation. You know that one meets with these germs on the skin, in the digestive tubes, in the regions of the organism in communication with the air, in those parts which, according to the expressions of Claude Bernard, continue to belong to the exterior world. You succeed in catching a glimpse of the processes capable of opposing the passage of these microbes from the cutaneous or mucus surfaces into the tissues. Thus the positive, solid, and sure ideas enabling the danger to be avoided are already numerous and clearly codified. This is not the time, therefore, to leave a free career to the ignorant; still less is it time when to these theoretical ideas are added practical data! Antiseptics, or to speak less pretentiously, cleanliness—external, internal, and scientific—here are your grand means! You are even taught to examine the working of certain machinery—such as the kidneys, for instance; you are taught how to seek for albumen, to advise a milk diet, guiding yourselves on pathological symptoms; to see, above all, that the doctor is called in without delay."

In conclusion, Doctor Charrin thus apostrophised the new midwives as to their all-important mission: "You will be the apostles of hygiene, of cleanliness in every way; you will fight against prejudices; everywhere you will bring in the sun, the light, those great hygienists; you will ventilate those rooms where the sick and the well are crowded together, even to poisoning each other; you will set yourself against all alimentation that is too solid, too soon administered to the delicate digestive tube of the newly-born; for the newly-born, as well as the newly-confined woman, in some measure belongs to you. Doubtless you should not forget the limits imposed on your intervention by the wisdom of the law—wisdom, which truly is not immutable! But in the sphere reserved to your activity you can do much; you can by isolation avoid contagion; you can perform healthy vaccinations, especially in localities refractory to this practice; you can, as regards wet nurses, give excellent advice, ward off many serious troubles; you can, remembering the notions of pathology taught you here, prevent, or even cure, many illnesses by having the doctor called betimes for respiratory, digestive, &c., affections. In other ways, you would be able to reassure a sorrowful mother in distress at the sight of her baby in convulsions, the ill renown of which, alas! is frequently justified by their connection with the fatal meningitis. You will end these convulsions, sometimes accidental, by ascertaining the cause; you will do so, according to circumstances, by soothing the teething, by expelling a tapeworm, by removing a pin or any other cause which by irritating the skin, flesh, or mucus, could produce reflex processes."

EDMUND R. SPEARMAN.

### Where to Go.

MESSRS. HARRIS'S ART NEEDLEWORK EXHIBITION, 25, BOND STREET, W.—It is a great relief to the monotony of the sick-room, especially in cases of tedious convalescence, to have pretty needlework on hand, and nurses who like such cannot fail to appreciate a visit to this exhibition. The materials used are made entirely of flax or flax woven with silk, and have the advantages of being durable, washable, and beautiful. The designs are artistic, some being bold and effective, others delicate and fine. The articles are numerous, from portières and curtains to book covers, splashers, tea cosies, tablecloths, d'oyleys, tidies, &c. One novelty is the "Pick Up" tidy. It is a large square of linen, lined, with a pocket in the middle for materials; the work remains on it, and when it is necessary to lay it aside the four corners are lapped over, one being prettily embroidered with an appropriate design. Tea cosy and tea cloth, embroidered to match, are charming, and as both are easily washed they can be kept daintily fresh. "The Willow" pattern is a very clever design, and it is easily worked, as the stitches are as simple as they are effective. It is impossible to mention the host of pretty things that are on show for this season, but nurses in charge of infectious cases are reminded that as these materials wash, and are thereby disinfected, they are most suitable for them. The flax and silk cloth is useful for tea gowns, &c., the linen is finding favour amongst nurses for dresses, and cycling dresses of the same material are becoming popular.

### Minor Appointments.

ENFIELD COTTAGE HOSPITAL.—On June 2nd Miss E. S. Boyer was appointed Nurse-Matron of this hospital out of 100 applicants. She was trained for one year at the Children's Hospital, Cromwell Road, Highgate, in connection with the Great Ormond Street Hospital for Sick Children, and holds a three-year certificate from the General Hospital, Cardiff, and one for six months from the Eastern Fever Hospital, Homerton, N.E.

ST. MARY'S HOSPITAL, MANCHESTER.—Miss Rose Merritt, who was trained at and afterwards on the private staff of Guy's Hospital, was recently appointed Assistant Matron here.

THE PARK HOSPITAL, LEWISHAM.—Nurse Britton, who recently completed her three years' training at Guy's Hospital, has been appointed Charge Nurse of this hospital.

WOLVERHAMPTON WORKHOUSE.—Miss Maud Carter, who was trained at Brownlow Hill, was appointed Superintendent Nurse on June 3rd.

### Everybody's Opinion.

[Correspondence on all subjects is invited, but we cannot in any way be responsible for the opinions expressed by our correspondents. No communication can be entertained if the name and address of the correspondent is not given, as a guarantee of good faith but not necessarily for publication, or unless one side of the paper only is written on.]

### COTTAGE HOSPITAL.

F. BRUMELL, jun., Morpeth, writes: My district nursing committee are thinking of beginning a small cottage hospital by providing a few beds at their nurses' home for cases requiring special care, the nurse to continue her district nursing, and with the aid of an assistant to attend to the cases in hospital. Available income £200 a year. If any reader acquainted with an organisation on these lines will be kind enough to send me a copy of the scheme and rules I shall be greatly obliged.

### NURSING IN WEST AFRICA.

"A NURSING SISTER IN WEST AFRICA" writes: I have been away from home and did not see Miss Kingsley's letter until this week, or should have answered it before. I only saw part of Miss Kingsley's lecture that was published in THE HOSPITAL, and certainly there was no mention of the hospital at Accra. I quite admit more hospitals and nurses are wanted on the coast, but cannot agree with Miss Kingsley's idea of the hospital ships, as there are certainly too many drawbacks at present. Ships to be outside the fever belt must lie three miles from shore, and whatever may be said I don't believe one man in a hundred would go willingly that distance in a surf boat. Again, I have always found patients thankful to get off the steamers and into hospital, even when they have only come the short distance from Cape Coast Castle. I am speaking of Accra, as that is the place I know best, and I think I am right in saying that, with the exception of Adah, we have the worst surf on the coast. I hope to go out again shortly, and if Miss Kingsley or anyone can give me any help or ideas I shall be very glad; and I may mention here that we are trying to form a small library, and should anyone feel inclined to give books I will willingly take them out with me.

### DIET CHART.

"S. A. M." writes: Seeing in THE HOSPITAL Nursing Mirror for May 28th that "Vincit Veritas" would like to know if there is an infants' diet chart published giving the number of feeds in twenty-four hours, and the quantity according to age, I should so much like her to know of a little book that has been a very great help to me, called "Our Baby," for mothers and nurses, by Mrs. Langton Hewer (Hirschfeld Bros., 82, High Holborn). I do not know if you have room in your paper for the following table, but "Vincit Veritas" can get the whole book for 1s., and it is full of interest and instruction from cover to cover:—

| Age. | Meals. | Cow's Milk. | Barley Water. | Each Meal. | Total. |
|---|---|---|---|---|---|
| 1 week | 10 | ½ oz. | 1½ oz. | 2 oz. | 20 oz. |
| 1 month | 9 | 1½ oz. | 2½ oz. | 4 oz. | 36 oz. |
| 2 ,, | 8 | 3 oz. | 3 oz. | 6 oz. | 48 oz. |
| 3 ,, | 8 | 3½ oz. | 2½ oz. | 6 oz. | 48 oz. |
| 4 ,, | 8 | 4 oz. | 3 oz. | 7 oz. | 56 oz. |
| 5 ,, | 7 | 5 oz. | 3 oz. | 8 oz. | 56 oz. |
| 6 ,, | 7 | 6 oz. | 2 oz. | 8 oz. | 56 oz. |
| 7 ,, | 7 | 7 oz. | 2 oz. | 9 oz. | 63 oz. |
| 8 ,, | 7 | 8 oz. | 1 oz. | 9 oz. | 63 oz. |
| 9 ,, | 6 | 9 or 10 oz | — | 9 or 10 oz. | 54 or 60 oz. |
| 10 ,, | 6 | 9 or 10 oz. | — | 9 or 10 oz. | 54 or 60 oz. |

If "Vincit Veritas" wants to make a complete study of the subject she should read "Artificial Feeding and Food Disorders of Infants," by W. B. Cheadle, M.A., M.D., Cantab., F.R.C.S. (Smith, Elder, and Co., 15, Waterloo Place, London).

### A Book and its Story.

"HIS FORTUNATE GRACE."

The theme of this little story,* which we commend to the notice of our readers, is based on the Anglo-American alliances which of late years have flooded the marriage market, and it is readily recognised that the plot, for the most part, is based on real life. The scene of the story is laid in America, and it opens at a breakfast table in New York, where the wealthy Mr. Forbes, of that city, and his daughter are discussing certain financial questions which narrowly concern the young lady's future. Miss Forbes is described as being "not handsome; her features were large and her complexion dull; but she had the carriage and 'air' of the New York girl of fashion, and wore a French morning toilette which would have ameliorated a Gorgon." If Miss Forbes was lacking in personal beauty she was not lacking in all attributes which go to make an interesting personality —she was heiress of many millions, and she had a "mission." It was as champion of a cause that the reader is first introduced to Augusta Forbes. In this opening chapter Miss Augusta informs her father that she would wish him to divide his fortune with his fellow men. "It is terrible," she exclaims, "terrible to think of the starving thousands. I feel it my duty to tell you, papa, that if you do not do this yourself I shall—when—when—but I cannot even think of that." "Suppose you fall in love and want to marry?" suggested Mr. Forbes. "I shall tell him everything—what I intend to make of my life, do with what wealth I have at my disposal. If he does not sympathise with me and agree to my plans, he must go. A woman's chief end is not matrimony." This Miss Augusta Forbes, aged 21, was an advanced young woman, immersed to a serious degree with questions of political economy and the distribution of wealth. She belonged to a club of young girls among the upper ten of New York, who aspired to be "something more than fashionable butterflies." That Miss Forbes was not wholly consistent in her unselfish theories towards the people—the starving thousands—is subtly shown in succeeding pages.

Neither her father nor her mother were in sympathy with her earnestness, but the members of the Girls' Club were one with her, and that sufficed, and "she was quite happy. She had never known an ungratified wish; she was spoken of in the newspapers as one of the few intellectual young women in New York society, and now she had a really serious object in life. She felt little spasms of gratification that she had been born to set the world to rights—she and a few others."

The meetings of these social reformers, great in their own estimation, are amusingly described at full length in the pages which succeed the first chapter.

A new element in the story comes with the arrival on the scene of the Duke of Bosworth, an impoverished young Englishman, who has crossed to America with the more or less avowed object of replenishing his exchequer through the acquisition of a wealthy wife. His Grace was a small and delicately-built man of 30 or more. "His cheeks and lower lip were beginning to droop. The pale blue eyes were dim, the lids red. He was . . . 'a good sort,' and men liked him."

Somewhere on the Continent the Duke had come across a smart young American woman by name Miss Mabel Creighton, who figures in these pages as another female champion of "the people"—she was one of the "club girls" to whom Augusta Forbes had referred. Away from her home surroundings, the Duke had been much taken with Miss Creighton's piquancy, and with her babyish voice and her delightful manner; he now crosses the ocean and visits her at her own home. Miss Creighton was very rich.

Rapidly succeeding his Grace's re-introduction to the object of his matrimonial intentions, an engagement is entered into between the two, to be as suddenly broken off upon the wreckage of the fortunes of Creighton père.

Mabel Creighton and Augusta Forbes were friends. Upon learning of the broken engagement the latter paid a visit to the former, and with some precipitation put the following question: "Mabel, should you think it dishonourable if I married the Duke of Bosworth?" to which question the other replied, "If I did, would it make any difference?" "No, but I'd rather you didn't . . . as you cannot marry him yourself. I don't see why I shouldn't. . . ." Mabel turned her head and regarded Miss Forbes with a haughty stare. "I do not love him," she said; "I despise him too thoroughly. It is my pride only that is violated. Don't let there be any doubt on that point." After this the attentions of his Grace are centred on Miss Forbes, but the course of true love never yet ran smooth, and Augusta's father gives but scanty encouragement to the match. This is the most interesting part of the story, and Miss Atherton displays her skill in the delineation of the characters of the parents of the headstrong Augusta. At this point in the narrative once more father and daughter faced each other across the breakfast table. This time Augusta, with a very red face, stared defiantly into bitter and contemptuous eyes. The conversation of the two turned on the consistency of the daughter's former wishes as regarded the distribution of her wealth. "And your Socialism? Do you expect to convert your Duke?" asks Mr. Forbes. "No, papa, of course not." "It is exactly five weeks since you informed me you wished to devote my fortune to the dear people." "I know it, papa. One looks at things very differently when one looks at them through a man's eyes, as it were. . . . Now it seems to me a grand thing to restore the fortunes of an ancient and illustrious family." Mrs. Forbes is a personality admirably portrayed in lifelike terms. A beauty, married at an early age to an adoring husband, she is yet a beautiful woman at 38. She fills the position of a queen in New York society. Her power was great—"New York *c'est moi*"—and she was aware of it. His Grace has a warm champion in Augusta's mother, whose ambition alienates her temporarily from the affections of her adored and adoring husband. At all costs the marriage, she determines, is to take place, and for that reason she sails for England, taking her daughter with her, and later the marriage does take place—in the country of his fortunate Grace. The mother has effected what she regards as her duty to her child, and this work accomplished, the wife's love for the husband overpowers her, and, in response to her cable of entreaty for his presence, before the wedding day, he had arrived in London.

When the ceremony was over, and Mr. and Mrs. Forbes had returned to the hotel, she put her arms on his shoulders and looked him in the eyes.

"Tell me," she said, imperiously, "have you really forgiven me? I have almost been sure at times that you had. I have felt it. But you have not been quite your old dear self. I want to hear you say again that you forgive me."

"Yes," he said, "I have forgiven you, of course. We are to live the rest of our lives together. I am not so unwise, I hope, as to nurse offended pride and resentment."

There is power and pathos in the manner in which the authoress handles the last chapter of her story, which terminates with the repentant woman's words, "Tell me," she said to her husband, her voice vibrating, "won't it ever be quite the same again? Is that what you mean?" He took her in his arms and laid his cheek against hers. "Oh, I don't know," he said; "I don't know."

* "His Fortunate Grace." Gertrude Atherton. (London: Bliss, Sands, and Co.)

### For Reading to the Sick.

"WORKERS TOGETHER WITH HIM."

**Verses.**

ONLY work that is for God alone
Hath an increasing guerdon of delight,
A guerdon unaffected by the sight
Of great success, nor by its loss o'erthrown,
All else is vanity beneath the sun,
There may be joy in doing, but it palls when done.
—*F. R. H.*

We may do
Our Father's business in these temples murk,
Thus steadfast, thus intent and strong;
While thus, apart from toil our souls pursue
Some high calm spheric tune, and prove our work
The better for the sweetness of our song.—
*E. B. Browning.*

God asks not what, but whence thy work is from the fruit,
He turns His eye away, to prove the inmost root.
—*Trench.*

Not stirring words, nor gallant deeds alone,
Plain patient work fulfilled that length of life;
Duty, not glory—service, not a throne,
Inspired his effort, set for him the strife. —*Clough.*

Art thou alone? and does thy soul complain
It lives in vain?
Not vainly does he live who can endure!
O, be thou sure
That he who hopes and suffers here, can earn
A sure return!
Hast thou found naught within thy troubled life
Save inward strife?
Hast thou found all she promised thee, Deceit,
And Hope a cheat?
Endure! and there shall dawn within thy breast
Eternal rest! —*A. Proctor.*

**Reading.**

Every man's task is his life preserver. The conviction that his work is dear to God and cannot be spared defends him.—*Emerson.*

"Every man is wanted, but no man is wanted much." When we have learnt the nothingness of our work—but not till then—we need to learn its value. What makes it pure gold is its conformity with God's will. What He wants done must be done, and will be done; and, if we are privileged to do any part of it, let us thank Him for the greatest mercy of this life. Worth while a thousand years of life, if by one word or deed we can help to build God's City; but let us remember that we know not the place or the value of the stones of that building—only the Master Builder knows this—it is our part to labour diligently in bringing stones, praying Him to use them. It may be that more important issues, in the great spiritual combat, cluster round "some daily strife," or some struggle of patience on a sick bed, than round apparently important posts. Our feeling should not be, "God is so mighty I need not work," but, rather, "I am part of so tremendous a battle, and the issues are so confused that every trifle in my daily life may be the key of some important position." He to Whom time is as eternity and eternity as time is delivered from all strife. The thought, "time is as eternity," makes us feel the littleness of the greatest earthly interests; but the converse, "eternity is as time," throws another light on all around us, and reveals the interests of eternity wrapped up in the things of time, whether great or small.—*Lucy H. M. Soulsby.*

### Notes and Queries.

*Professional Dress.*

(98) Would it be considered correct for a nurse travelling for pleasure to wear her uniform at dinner?—*Peek-Frean.*

No. It is not advisable to wear professional dress on a pleasure trip. There is a feeling that it is better to wear ordinary dress when entirely away from duty; but during the intervals of duty, even when off, say, for a day, uniform is quite correct.

*Blankets.*

(99) Is it usual for blankets used by patients and staff in the London hospitals to be washed by the nurses?—*C. I. M.*

Most certainly not. A nurse is not a laundress.

*Edinburgh Schools for Dispensing.*

(100) Would you tell me where I could learn dispensing in Edinburgh, and what would be the fee?—*Maud Violet.*

A lady can learn dispensing at the Royal, the New Town, or the Cowgate Dispensaries, the fee for attendance being £1 a month. Three months' attendance at least would be necessary, and it would be most advisable to take, at the same time, a course of theoretical and practical materia medica at the Medical College for Women, 20, Chambers Street, Edinburgh. The lecturer for these is Dr. W. Craig, and the class fees would be £3 5s. for the lectures and £3 3s. for the practical instruction.

*The Treatment of Burns.*

(101) Will you kindly tell of the best way to apply picric acid as treatment for a burn—the mode of dressing and the strength of acid? Is it only of use in case of a recent burn?—*A. H. W.*

Picric acid is used in the treatment of recent burns in which the whole thickness of the epithelium has not been entirely destroyed. It is employed as a saturated solution. The surface is first washed with some efficient antiseptic, the blisters are opened and the contained serum let out, and then a piece of lint dipped in a saturated solution of picric acid, made with sterilised water, is placed over the injured part and is covered with a thick layer of sterilised absorbent wool. The pain produced soon passes off. The dressing becomes a dry one, and is left on for three or four days.

*How to Become a Doctor.*

(102) "Own Earnings" asks how a young chemist can become a doctor, and "what course of self studies to pursue at a considerably cheap rate."

The Registrar of the General Medical Council will give particulars as to the examination in general education which is required, and the dean of any medical school will, no doubt, be very glad to forward a prospectus giving the course of study which must be followed afterwards. The first thing is to pass the entrance examination.

*Lupus.*

(103) Will you kindly tell me through your columns whether the skin disease, Lupus erythematosus is serious or dangerous?—*A. H. H.*

Lupus erythematosus is a serious malady, in the sense that it is difficult to cure and tends to leave a scar. Consult a specialist, *i.e.*, a physician attached to the skin department at one of the great hospitals.

*Nursing in Cairo.*

(104) "H. C. F." is in error in supposing there are any private nurses attached to Kars-et-Aini Hospital, which is strictly a hospital for natives: There are three nurses for private work employed by the committee of the Cairo Sick Nursing Fund, supported by subscriptions and the nurses' fees, of which Dr. Sandwith is secretary, but this fund has nothing to do with Kars-et-Aini or any other hospital. There is no private nursing institution in Cairo. If "Ra[illegible]k" wishes to work there she should go out in December or January, and find lodgings at a boarding house there for about three or four months. She stands a very good chance of work, and probably a passage home with a patient at the end of that time. Board and lodging in Cairo at the cheapest boarding house will cost two guineas a week, and personal washing is 2s. 6d. per dozen articles.—*H. T.*

*Holiday Homes.*

(105) "Mimi" asks for addresses of nurses or students' holiday homes in Kent or Sussex, not sea-coast, country preferred.

See "Homes and Hospitals for the Benefit of Gentlewomen," 1s., from the Army and Navy Co-operative Society, Victoria Street, Westminster.

*California for Consumption.*

(106) (1) Can you tell me of a suitable place in California for phthisical cases? (2) Also if there is an opening for English nurses?—*Kingham.*

The first question can only be satisfactorily answered by the medical man in charge of the patient. If it be worth while to go to California for health it is worth while to consult a specialist as to the particular locality in which he or she will derive most benefit. (2) There is no demand for English nurses in America, but a well qualified woman able to bide her time will undoubtedly be able to earn her living. The fees for nurses in San Francisco run from 20 to 25 dols. a week. Miss Elsie Wallace, the lady superintendent of the Training School for Nurses, 3,700, California Street, San Francisco, might possibly give you some help.

*School for Imbeciles.*

(107) Kindly tell me to whom to apply for information of vacancies for probationers at Schools for Imbecile Children Darenth. Is Newcastle Royal Infirmary considered a good training school for nurses?—*A Constant Reader.*

Apply to the Matron, the School for Imbecile Children, Darenth, Dartford, for particulars. (2) Newcastle Royal Infirmary ranks as one of the larger provincial training schools. It possesses 270 beds, and has a large staff of nurses. Candidates are required to undergo a three months' trial before becoming probationers. There is no salary for the first year.

